# Wafer-scale integration of GaAs/AlGaAs core–shell nanowires on silicon by the single process of self-catalyzed molecular beam epitaxy

**DOI:** 10.1039/d2na00848c

**Published:** 2023-01-23

**Authors:** Keisuke Minehisa, Ryo Murakami, Hidetoshi Hashimoto, Kaito Nakama, Kenta Sakaguchi, Rikuo Tsutsumi, Takeru Tanigawa, Mitsuki Yukimune, Kazuki Nagashima, Takeshi Yanagida, Shino Sato, Satoshi Hiura, Akihiro Murayama, Fumitaro Ishikawa

**Affiliations:** a Research Center for Integrated Quantum Electronics, Hokkaido University Sapporo 060-0813 Japan ishikawa.fumitaro@rciqe.hokudai.ac.jp; b Faculty of Information Science and Technology, Hokkaido University Sapporo 060-0814 Japan; c Graduate School of Science and Engineering, Ehime University Matsuyama 790-8577 Japan; d Graduate School of Engineering, The University of Tokyo 113-8656 Japan

## Abstract

GaAs/AlGaAs core–shell nanowires, typically having 250 nm diameter and 6 μm length, were grown on 2-inch Si wafers by the single process of molecular beam epitaxy using constituent Ga-induced self-catalysed vapor–liquid–solid growth. The growth was carried out without specific pre-treatment such as film deposition, patterning, and etching. The outermost Al-rich AlGaAs shells form a native oxide surface protection layer, which provides efficient passivation with elongated carrier lifetime. The 2-inch Si substrate sample exhibits a dark-colored feature due to the light absorption of the nanowires where the reflectance in the visible wavelengths is less than 2%. Homogeneous and optically luminescent and adsorptive GaAs-related core–shell nanowires were prepared over the wafer, showing the prospect for large-volume III–V heterostructure devices available with this approach as complementary device technologies for integration with silicon.

## Abstract 2

Optically efficient GaAs/AlGaAs core–shell nanowires on 2-inch Si wafers before (front right) and after (others) growth. The samples show a dark-colored feature indicating light absorption on the substrate surface.

## Graphical abstract

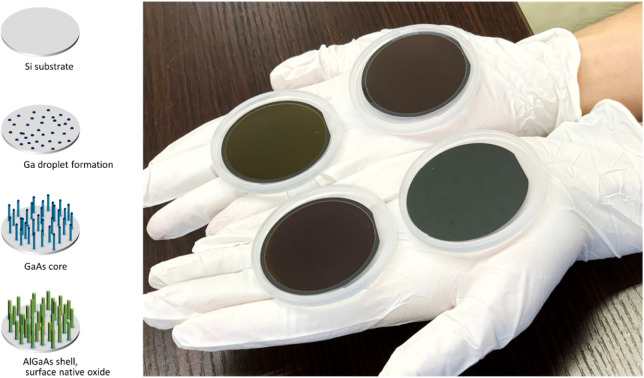

## 1. Introduction

The integration of materials having efficient optical functions with Si electronics has been pursued for decades,^1,2^ and the integration of III–V semiconductors provides superior electronic and optical functions to the developed Si technology.^3–5^ Among these semiconductors, the epitaxial growth of III–V semiconductor nanowires has been developed for practical applications^6,7^ requiring large output, such as energy-conversion systems, photovoltaics,^8^ large-scale production, and electron–photon conversion efficiency.^9^ The integration of III–V semiconductors into mainstream Si electronics has been pursued by various approaches, such as deposition, epitaxy, and direct wafer bonding.^5,10,11^ The epitaxial growth of III–V semiconductors on Si is difficult due to having different crystal structures and thermal expansion coefficients. Planar thin-film growth of III–V materials on Si has been performed with buffer layers, growth on patterned Si surfaces, or selected area growth from small openings resulting in optical devices with III–V functionalities.^12,13^ Advanced epitaxial techniques for nanowires can overcome these difficulties with III–V semiconductors and Si using heteroepitaxial growth.^14^ The growth mechanism of the nanowires allows for sensitive control of the nanowire dimensions, crystal structure, and material composition with possible control of the doping and heterostructural design.^15,16^ Therefore, the use of III–V nanowires on versatile Si substrates offers a realistic prospect for large-scale integrated systems with superior electronic and optical functions.^7,17^ Mass productivity and application at the macroscale are essential to obtain large output for the realization of highly efficient nanowire-based energy-conversion devices such as solar cells, photovoltaics, and photocatalysts.^18,19^ Metalorganic vapor-phase epitaxy, molecular beam epitaxy (MBE), and chemical-beam epitaxy are the common and realistic methods to produce III–V nanowires and obtain desirable properties.^18^ However, these methods are typically considered to be non-cost effective due to the growth capacity.^18^ Approaches to overcome the issue of large-volume nanowire production are limited, such as the use of an aerosol-based gas-phase growth method.^20^ In this report, we show that self-catalyzed MBE can overcome difficulties in the large-volume production of high-quality nanowires on Si wafers which is realized by the optimization of growth conditions. We demonstrate a rational approach to provide 2-inch wafer-scale III–V GaAs nanowires on Si(111) substrates using constituent Ga-induced self-catalyzed MBE with bright and homogeneous photoluminescence properties at room temperature.

## 2. Results and discussion

Ga-induced self-catalyzed vapor–liquid–solid nucleation and the growth of GaAs nanowires do not require an external catalyst such as the commonly employed Au.^21^ Therefore, growth can be performed without any pretreatment of the standard epi-ready Si substrate. This enables the growth of large-volume GaAs nanowires over the substrate wafers as cost-effective energy-conversion materials by a simple and cost-effective approach. Therefore, growth was conducted by MBE on phosphorus-doped n-type Si(111) substrates using constituent Ga-induced self-catalyzed vapor–liquid–solid growth. An epi-ready commercial Si substrate was introduced into the growth chamber and growth was started after 40 min of thermal treatment at 600 °C. Optimization of growth conditions, such as the substrate temperature, rate of flux, and the V/III ratio in the initial growth stage of the GaAs core, is effective for obtaining dense nanowires over the substrate. The appropriate growth conditions employed to obtain dense and high-quality nanowires over a 2-inch Si wafer were a Ga flux of 6 × 10^14^ cm^−2^ s^−1^, an atomic Ga : As_4_ flux ratio of 1, and a substrate temperature of 500 °C.

**Fig. 1:**
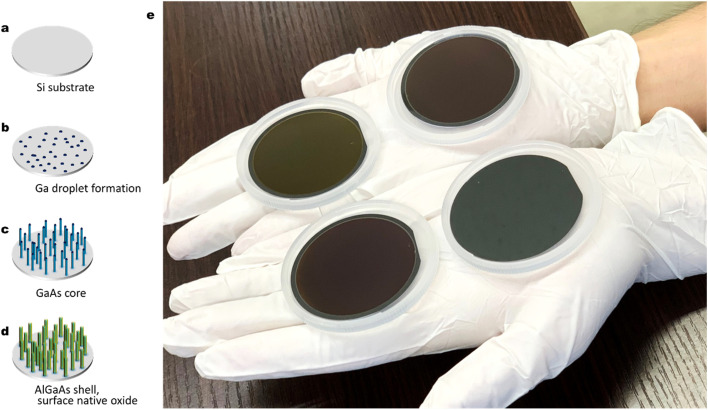
(a)–(d) Procedure for the formation of nanowires on a Si substrate. (e) 2-Inch Si wafers before (front right) and after (others) nanowire growth. Samples after nanowire growth show a dark-colored feature indicating light absorption on the substrate surface, except for the periphery where the substrate was held during growth. The slightly different colors of the nanowire samples reflect the slight difference in the Al composition and thickness of the outermost AlGaO_*x*_ shells for each nanowire sample.

Fig. 1(a)–(d) show the formation procedure of GaAs/AlGaAs core–multishell nanowires on a Si substrate and Fig. 1(e) shows a comparison of a Si wafer before and after the nanowire growth. The dark area containing dense nanowires has higher light absorption properties. This feature is different from that of the initial Si wafer before growth. The spread of this dark feature was observed over the entire substrate surface for the three series of samples shown in Fig. 1, except for the edge of the substrate, where the substrate holder interrupts the molecular beam flux during growth. The slightly different colors observed in the wafer surfaces between the samples are probably due to 80–87% differences in the Al composition in the AlGaAs outermost shells, which results in sensitive differences due to deviations of the refractive index of the outermost AlGaO_*x*_ native oxide within those samples.^22^

**Fig. 2:**
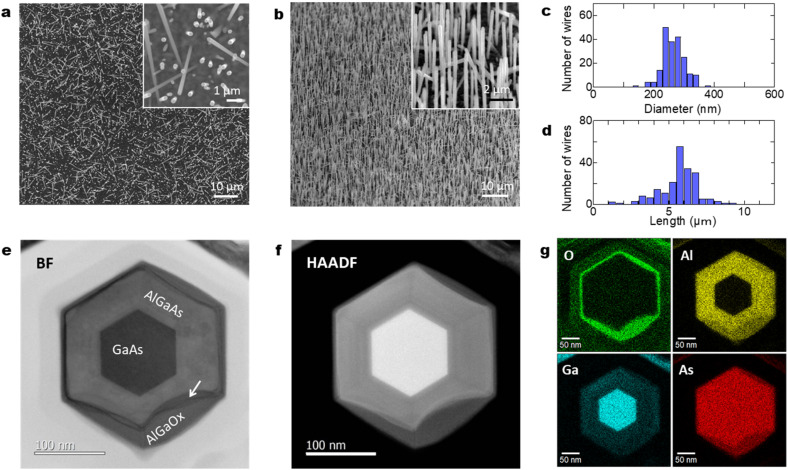
(a) Planar surface SEM image of GaAs/Al_0.8_Ga_0.2_As core–shell nanowires and higher magnification image (inset), (b) 45°-tilted SEM surface image and higher magnification image (inset) and distributions of the (c) diameters and (d) lengths of those nanowires. Axial cross-sectional (e) BF-STEM and (f) HAADF-STEM images of a typical nanowire. (g) EDS elemental maps for O, Al, Ga, and As. The arrow in (e) indicates the interface structure between the original AlGaAs shell and the oxidized AlGaO_*x*_.

Fig. 2(a) shows planar scanning electron microscopy (SEM) images of GaAs/Al_0.8_Ga_0.2_As core–shell nanowires at different magnifications. The nanowires have a density of 5 × 10^7^ cm^−2^ with a vertical yield of approximately 85%. The length and diameter of the nanowires were *ca.* 6 μm and 250 nm, respectively. The nanowires have a hexagonal cross-section, as shown in the planar SEM image presented in the inset of Fig. 2(a). Fig. 2(b) shows 45°-tilted SEM images of a sample under different magnifications, where dense nanowires are present on the surface. Surface passivation is a critical issue in the use of GaAs nanowires in practical applications because GaAs suffers from non-radiative surface recombination that is several orders of magnitude greater than those of most other III–V semiconductors such as InP and GaN.^23^ GaAs/AlGaAs core–multishell nanowire systems with AlGaAs barriers for carrier confinement are used to avoid the undesired surface effects. The core–shell nanowires enable tuning of the properties and provide passivation for core material surfaces, which results in an extensive range of high-performance device applications.^24^ On the other hand, it has been recently reported that an Al_2_O_3_ oxide shell passivation acts as an efficient passivation shell for nanowires.^25^ There is a mature oxidation technique for III–V optical devices which could be applicable to nanowires, such as laser processing to obtain electrical and optical confinement due to the stability of the oxides.^26^ We recently employed a simple passivation method where an Al-rich AlGaAs outer shell forms a native oxide of (Al,Ga)O_*x*_ at the surface by exposure to the ambient atmosphere, which provides stronger electrical and optical confinement in the GaAs nanowire core and stability of the oxides.^27^Fig. 2(c) and (d) show the distribution of the wire diameter and length of the 200 nanowires investigated. The wires show a diameter of 262 nm with a standard deviation of 33 nm, and a length of 5.9 μm with a standard deviation of 0.62 μm.

Fig. 2(e) and (f) show axial cross-sectional scanning transmission electron microscopy (STEM) images of a GaAs/AlGaAs core–multishell nanowire acquired in the bright-field (BF) and high-angle annular dark-field (HAADF) mode. Fig. 2(g) shows energy-dispersive X-ray spectroscopy (EDS) elemental mapping distributions for Al, O, Ga, and As. The nanowire has a hexagonal cross-section with a 250 nm diameter. The nanowire consists of a regular hexagonal GaAs core with a 120 nm diameter surrounded by a 60 nm thick Al_0.8_Ga_0.2_As shell. The surface of the outermost AlGaAs shell was oxidized to form an AlGaO_*x*_ outer shell, as clearly shown by the EDS elemental maps in Fig. 2(g). The As concentration is low at the outer shell surface, whereas the concentration of Al and O is higher, indicating that exposure of the sample to the ambient air is sufficient to oxidize the outer Al-rich AlGaAs shell.^26,27^ A dark contrasted area is observed at the interface between the initial AlGaAs shell and the oxidized AlGaO_*x*_, as indicated by the arrow in Fig. 2(e). This area was more obvious in the strain-sensitive BF image in Fig. 2(e) but weaker in the composition-sensitive HAADF-mode *Z*-contrasted image in Fig. 2(f). This result shows that the interface has strain accumulation induced by the difference in the lattice structure between AlGaAs and AlGaO_*x*_. The HAADF image and EDS maps indicate that the strain is accumulated mainly in AlGaAs and the interface is considered to be deformed by the neighboring AlGaO_*x*_ oxide. The nanowires were stable for over one year because the measurement was carried out more than a year after the growth of the nanowires. The extent of oxidation from the surface varies from nanowire to nanowire (see Sections 4.6 and 4.7 in the following), which is likely related to the structural variations between the nanowires. Overall, throughout the nanowires on the sample, the inside of the AlGaAs shell and the inner GaAs core showed negligible oxide concentrations, which indicates that they are not affected by oxidation from the surface and the crystalline quality is preserved.

**Fig. 3:**
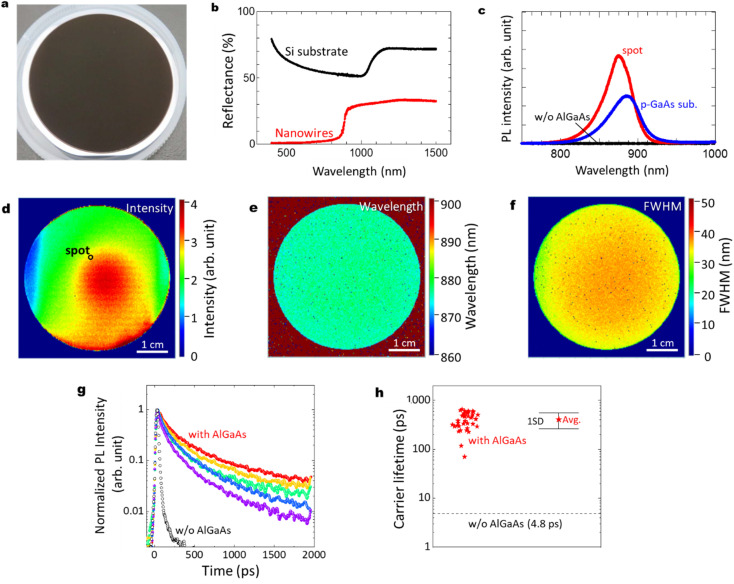
(a) Photograph of a 2-inch Si wafer after the growth of GaAs/Al_0.8_Ga_0.2_As core–shell nanowires. (b) Reflectance spectra from ultraviolet to near-infrared wavelengths for the pristine Si substrate and the sample after the GaAs/Al_0.8_Ga_0.2_As core–shell nanowire growth, (c) RT PL intensity for GaAs/Al_0.8_Ga_0.2_As core–shell nanowires, GaAs nanowires without the shell layers, and a commercial p-type GaAs substrate as a reference. PL mapping results for the GaAs/Al_0.8_Ga_0.2_As core–shell nanowires showing their distributions of (d) intensity, (e) wavelength, and (f) FWHM. (g) Normalized RT PL decay curves at different positions of the nanowires with the Al_0.8_Ga_0.2_As shell compared to that without the Al_0.8_Ga_0.2_As shell. (h) RT carrier lifetime measured at 40 positions of the nanowires with the Al_0.8_Ga_0.2_As shell, compared to that without the AlGaAs shell. The average value and the standard deviation are also indicated.

The optical characteristics of the GaAs/Al_0.8_Ga_0.2_As core–shell nanowire sample are shown in Fig. 3(a)–(h). As shown in Fig. 3(a), the sample with GaAs/Al_0.8_Ga_0.2_As core–shell nanowires has a dark surface over the Si wafer. Reflectance spectra from ultraviolet to near-infrared wavelengths for the pristine Si substrate and the sample after the GaAs/Al_0.8_Ga_0.2_As core–shell nanowire growth are shown in Fig. 3(b). The Si substrate shows a standard feature with an absorption edge around 1100 nm. In contrast, the reflectance of the nanowires sample is less than 33% over the spectral range, which is much lower than that of the silicon wafer in the same spectral range. Moreover, the nanowire sample shows much smaller reflectance at the wavelength below 880 nm, which agrees well with the absorption of the GaAs direct bandgap transition. The nanowire samples show reflectance smaller than 2% below the wavelengths 710 nm, and less than 1% at wavelengths below 540 nm. These results can be attributed to the light scattering due to the structural features of the nanowires^28^ and the light absorption by the GaAs core in the nanowires.

Fig. 3(c) shows PL results at room temperature (RT) for the GaAs/Al_0.8_Ga_0.2_As core–shell nanowires sample measured at the spot on the substrate surface shown in Fig. 3(d), compared with a GaAs nanowire sample without the outer AlGaAs shell, and a commercial Zn-doped p-type GaAs substrate (AXT Inc., synthesized by the vertical gradient freeze method). The GaAs/Al_0.8_Ga_0.2_As core–shell nanowires sample showed the strongest PL intensity with a peak wavelength of 876 nm, while no PL peak was detected from the GaAs nanowires without the AlGaAs shell. The p-type GaAs substrate has a peak at 884 nm with comparable or weaker intensity than the GaAs/Al_0.8_Ga_0.2_As core–shell nanowire sample. The slightly shorter peak wavelength for the passivated nanowires compared to the p-GaAs substrate is considered to be due to the compressive strain induced by the surface oxides.^27^Fig. 3(d)–(f) show PL mapping results of the GaAs/Al_0.8_Ga_0.2_As core–shell nanowire sample, a GaAs nanowire sample without the AlGaAs shell, and a commercial p-type GaAs substrate, respectively, at RT. The distributions of the spectral peak intensity, wavelength, and full width at half maximum (FWHM) are shown for the GaAs/Al_0.8_Ga_0.2_As core–shell nanowires. The PL intensity of GaAs/Al_0.8_Ga_0.2_As core–shell nanowires varies within the surface, as shown in Fig. 3(d), where most of the area has stronger intensity than the p-GaAs substrate as compared in Fig. 3(c). The central area showed stronger PL intensity, whereas that at the edge was weaker. The distribution of the PL peak wavelength was homogeneous over the sample with a peak wavelength of 876 nm and a standard deviation as small as 3 nm. Similarly, the peak FWHM was 35 nm with a standard deviation of 4 nm.

Fig. 3(g) shows RT time-resolved PL decay curves at different positions of the nanowire sample with and without (w/o) the Al_0.8_Ga_0.2_As shell. Both samples show a superposition of the fast and slow decay components. This multiexponential PL decay feature is considered to be due to the discrepancies between the carrier lifetimes of the nanowires, as previously reported for a single nanowire.^24^ Although variations in the PL decay features among these data are evident, the PL decay times are much slower than the net PL decay time of 4.8 ps for the nanowires without the Al_0.8_Ga_0.2_As shell. The average carrier lifetime and the standard deviation from the data measured at 40 positions were 407 and 145 ps, respectively, as indicated in Fig. 3(h). Note that the slower decay time of *ca.* 1 ns after 400 ps is comparable to the long carrier lifetime reported for the GaAs/AlGaAs system.^29^ Therefore, the AlGaAs shell passivation increased the carrier lifetime of the nanowires by two orders of magnitude, which provided efficient passivation for the nanowires. The highly homogeneous and efficient PL over the 2-inch Si wafer holds promise with respect to application in various electronic and optical integrated devices at RT.

## 3. Summary

Self-catalyzed MBE was used as a rational, simple, and cost-effective approach to obtain 2-inch wafer-scale GaAs-related nanowires on Si(111) substrates that exhibit bright and homogeneous PL at RT. This was realized by the single procedure without specific pre-treatments such as patterning, film deposition, or etching of the Si substrate; 6 μm long and 250 nm diameter GaAs/AlGaAs core–shell nanowires with a nanowire density of 5 × 10^7^ cm^−2^ were homogeneously grown on Si(111) substrates. The resultant 2-inch Si substrate sample exhibits a dark-colored feature due to the existence of nanowires where the reflectance in the visible wavelengths is suppressed less than 2% by the light scattering and absorption of the nanowires. The sample also showed bright PL comparable to or stronger than p-type GaAs substrates. Nanowire arrays are expected to produce a photogenerated current equivalent to that of a planar thin film while using significantly less material.^30,31^ From the dark area observed on the substrate due to the dense nanowires, it can be expected that resonant light absorption is induced by the nanowires. We can also expect efficient energy conversion according to the PL results. This approach could provide a scalable methodology for the fabrication of large-area nanowire-based applications, especially for energy-conversion systems that require large output.^32^

## 4. Technical details

### 4.1. Growth methods

**Fig. 4:**
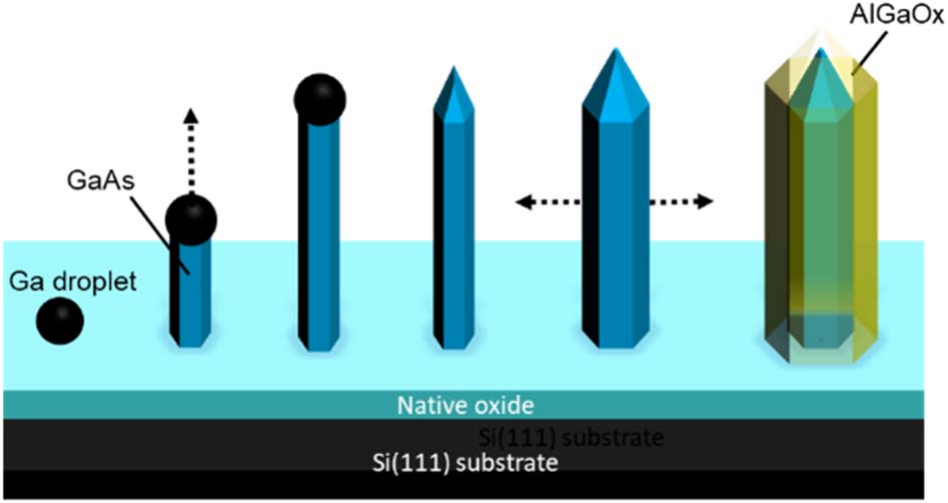
Schematic illustration of the procedure for the formation of GaAs/AlGaAs core–shell nanowires.

**Fig. 5:**
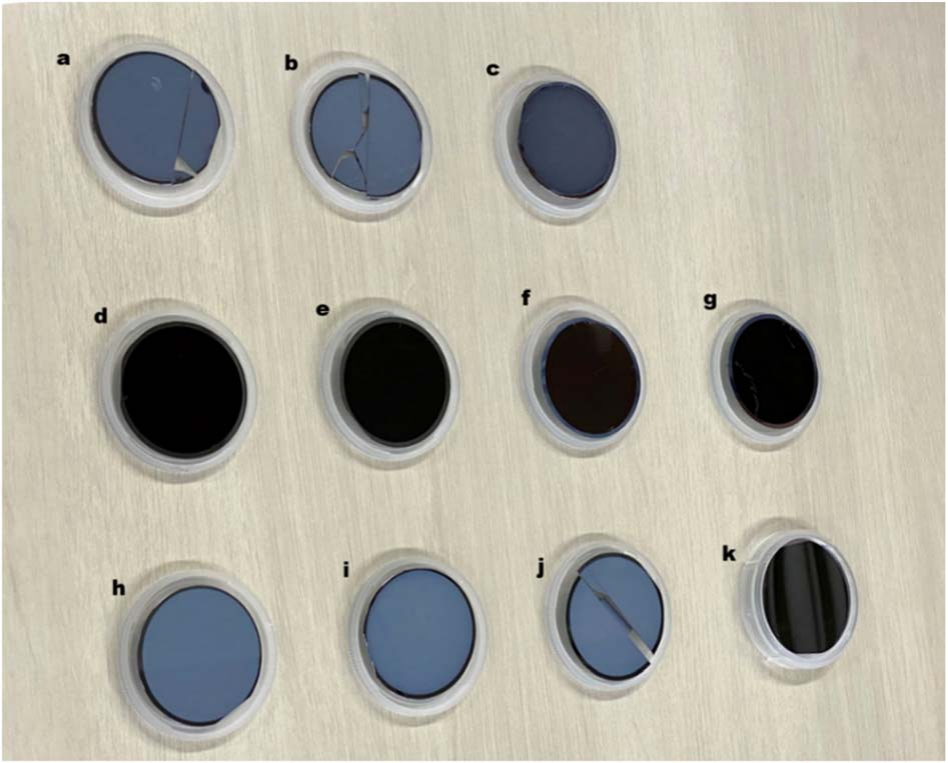
(a)–(c) Samples grown at different substrate temperatures of 540, 520, and 480 °C, respectively. The sample grown at a substrate temperature of 480 °C has a darker surface, which suggests that the substrate temperature has an effect on the surface characteristics. (d)–(g) Samples grown under identical growth conditions at a substrate temperature of 500 °C to examine the reproducibility of the growth process. (h)–(j) Samples grown at a substrate temperature of 560 °C with half the Ga and As_4_ fluxes to before, which correspond to 3.2 × 10^14^ cm^−2^ s^−1^, and variation of the growth conditions by (h) reducing the substrate temperature to 480 °C during the GaAs core growth. (i) Supply of catalyst Ga on the surface before initiating the GaAs core growth.^46^ (j) Reduction of the As supply to one-third during the core growth. (k) Si(111) substrate.

GaAs/Al_0.8_Ga_0.2_As core–shell nanowires were grown by MBE (RC2100, EpiQuest, Japan) using constituent Ga-induced self-catalyzed vapor–liquid–solid growth. Solid source effusion cells were used for Ga and Al. A valved cracker cell operating in As_4_ mode was used for As by adjusting the cracker zone temperature at 600 °C.^33,34^ The structure and composition-controlled AlGaAs multi-layered core–shell nanowire structure was formed using the standard MBE procedure involving shutter control and temperature adjustment of the Al and Ga cells while rotating the substrate.^21,27,33–35^ Epi-ready 2-inch phosphorus-doped n-type Si(111) substrates were used for nanowire growth. The Ga supply was set to obtain a planar growth with the rate of 1.0 monolayer/s on GaAs(001), which corresponds to a Ga flux of 6.3 × 10^14^ cm^−2^ s^−1^. The effective atomic flux ratio between the group-III Ga and group-V As_4_ beams was determined from the transition of the reflection high-energy electron diffraction patterns.^36^ The atomic V/III ratio was then adjusted to 1.0 by setting the As_4_ beam equivalent pressure to 6 × 10^−4^ Pa. The substrate was rotated throughout the growth process at 10 rpm. Before the growth process, the samples were heated to 600 °C for 40 min under an As_4_ atmosphere at a pressure of 6 × 10^−4^ Pa for thermal cleaning of the substrate surface. The substrate temperature was then reduced to 500 °C and GaAs core growth was initiated by opening the Ga shutter and allowing growth for 30 min.^36,37^ The examinations of growth conditions such as the substrate temperature, beam flux, and V/III ratio at the initial growth of GaAs core were determinants to obtain dense nanowires over the substrate (see Fig. 4 and 5 in the following).^37–42^

A schematic illustration of the procedure for the formation of GaAs/AlGaAs core–shell nanowires is shown in Fig. 4. Nanowire growth is initiated by Ga droplet formation on the native oxide-coated Si(111) substrate surface. This oxide layer presents pinholes, which form nanocraters that act as nucleation sites for the nanowires.^38^ The density of nanowires in an area is dependent on the substrate surface conditions. The wire predominantly starts to grow longitudinally for 30 min. Growth was interrupted to crystallize the Ga catalyst, during which the As_4_ pressure was increased to 8 × 10^−4^ Pa to promote lateral growth of the shell layers under group-V-rich growth conditions.^33–35^ After growth interruption, Al and Ga addition mostly resulted in lateral growth of the AlGaAs shell layer to produce the core–shell nanowires, which allows the 100 nm diameter nanowires to lengthen.^35^ After the growth of the GaAs shell layers for 10 min by opening the Ga shutter, the growth was again interrupted. The fluxes of Ga and Al were then set to 0.09 and 0.60 monolayers per s, respectively, which resulted in the growth of an AlGaAs layer with an Al composition of 80% within the group-III constituents. Growth was then re-started to form a 50 nm thick Al_0.8_Ga_0.2_As outermost layer over 45 min. This procedure was expected to form GaAs/Al_0.8_Ga_0.2_As core–shell structures with a total diameter of 250 nm, comprising a 130 nm wide GaAs core surrounded by a 55 nm wide Al_0.8_Ga_0.2_As shell. After the nanowire growth, the substrate temperature was reduced to 300 °C under an overpressure As_4_ atmosphere and the sample was then removed from the MBE chamber after cooling to ambient temperature. A series of samples were grown to examine the reproducibility of the samples. Three other samples were grown under almost identical growth conditions, which resulted in identical sample characteristics, except for a slightly different Al composition and width of the outermost AlGaAs shell layer; the variation in Al concentration was between 80% and 87%, and the variation in the AlGaAs shell width was within 50 to 60 nm. The outermost Al-rich AlGaAs shell in the nanowires was transformed into an oxide layer by native oxidation,^43^ which was performed by simply leaving the samples exposed to the atmosphere, typically for several days.^27^ The present investigation was conducted with samples that were exposed to ambient air for over one year. The growth procedures were conducted between December 2019 and October 2020, and scanning transmission electron microscopy (STEM) investigations were performed on February 2021, which demonstrates the stability of the samples.

### 4.2. Characterization methods

#### Structural characterization

The morphological properties of the nanowires were investigated using SEM (JSM-7001FA, JEOL, Japan). Cross-sectional STEM measurements on axially and radially sliced single nanowire samples prepared by focused ion beam (FIB) processing (Helios660, FEI, USA) were also performed; STEM measurements were conducted using a transmission electron microscopy system (JEM-ARM200F Dual-X, JEOL, Japan) operated at 200 kV to provide beam spot widths of 0.2 nm, and EDS measurements were performed with a 100 mm^2^ silicon drift detector (JED-2300, JEOL). Radial and axial cross-sectional STEM observations were performed for the FIB-processed specimens. Carbon is deposited to bury the nanowires for support. After carbon deposition, the specimens were etched to separate the original substrate. The specimens were picked up and attached to a FIB grid for further thinning of the film to a thickness of *ca.* 100 nm; STEM images were obtained in both BF and HAADF modes.^44^

X-ray diffraction (XRD) measurements were conducted with a Malvern Panalytical X'Pert MRD system equipped with a graded parabolic X-ray mirror with a four-bounce Ge(220) monochromator and Cu Kα_1_ radiation. Symmetric *θ*–2*θ* scans were performed with a three-bounce Ge(220) analyzer and a Xe proportional detector around the Si(111) reflection.

#### Optical characterization

A spectrophotometer (Shimadzu, Japan U-4100, SolidSpec-3700DUV) with an integrating sphere was used for obtaining the reflectance spectra of the nanowires sample from the visible to the near-infrared. A Halogen lamp was used as the light source, and the reflected light was collected with the photomultiplier tube below the wavelength 870 nm and InGaAs photodetector for the longer wavelengths. The spectra were calibrated with a white standard (Labsphere).

The PL mapping over the sample wafer was performed on a scanning microscope equipped with an *XY*-stage using a Horiba Jobin-Yvon LabRAM-HR system. The beam spot size was approximately 100 μm in diameter. The scan range was over an area of 50 × 50 mm with a step size of 250 μm. The measurement was performed using 532 nm excitation with a neodymium-doped yttrium aluminium garnet (Nd-YAG) laser with an excitation power of 0.1 mW. The measurements were conducted at room temperature (RT). The PL signal was collected using a 15× objective (NA = 0.3) with a backscattering configuration and detected with a thermoelectrically cooled CCD detector. As a reference luminescent sample, the PL intensity was compared with that of a commercial Zn-doped p-type GaAs substrate (AXT Inc.) synthesized by the vertical gradient freeze method.

Time-resolved PL was measured between 6 K and RT (295 K) using a streak camera (Hamamatsu Photonics, C10910-02) combined with a spectrometer *f* = 300 mm (Hamamatsu Photonics, C11119-04, 50 g mm^−1^ grating, 600 nm blaze). A mode-locked Ti:sapphire pulsed laser with a repetition rate of 80 MHz, a pulse width of less than 100 fs, and a spectral width of 10 nm was used as the excitation source. The instrument response function was *ca.* 14 ps at FWHM. Both the excitation and detection directions were almost normal to the sample plane.^45^ The laser wavelength was set at 750 nm to excite the GaAs nanowires, and the excitation power was set at 5 mW (*ca.* 50 W cm^−2^). The diameter of the excitation laser spot was approximately 100 μm, and thus the PL properties of the nanowires were obtained. The PL decay curves were fitted with a typical biexponential equation, *I*(*t*) = *A*_1_ exp(−*t*/*τ*_1_) + *A*_2_ exp(−*t*/*τ*_2_), where *τ*_1_ and *τ*_2_ represent the decay time constants, and *A*_1_ and *A*_2_ represent the amplitudes of each component. In this study, the average carrier lifetime was used to estimate the PL decay time *τ*_PL_, which can be defined as *τ*_PL_ = (*A*_1_*τ*_1_^2^ + *A*_2_*τ*_2_^2^)/(*A*_1_*τ*_1_ + *A*_2_*τ*_2_). The internal quantum efficiency (IQE) and the radiative and non-radiative recombination lifetimes of the nanowires were obtained from the temperature dependence of the PL intensity and *τ*_PL_, as described below. The nanowires were dense and the penetration depth of 750 nm excitation light was *ca.* 580 nm, which is sufficiently smaller than the nanowire length of 6 μm; therefore, the contribution of the parasitic layer on the substrate to the detected PL would be negligible. At low temperatures such as 6 K, the nonradiative lifetime of carriers (*τ*_nr_) is generally sufficiently longer than their radiative lifetime (*τ*_r_).^45^ Therefore, the IQE at 6 K can be simply assumed as 100%. In this case, the PL decay time (*τ*_PL_) can be defined as *τ*_PL_^−1^ = *τ*_r_^−1^ + *τ*_nr_^−1^. *τ*_r_ and *τ*_nr_ can be calculated using IQE(*T*) = *I*(*T*)/*I*(6 K) = *τ*_r_ − 1(*T*)/{*τ*_r_ − 1(*T*) + *τ*_nr_^−1^(*T*)} = *τ*_r_^−1^(*T*)/*τ*_PL_^−1^(*T*), *τ*_r_(*T*) = *τ*_PL_(*T*)/IQE(*T*) = *τ*_PL_(*T*)*I*(6 K)/*I*(*T*), and *τ*_nr_(*T*) = *τ*_PL_(*T*)/{1 − IQE(*T*)} = *τ*_PL_(*T*)*I*(6 K)/{*I*(6 K) − *I*(*T*)}, where *I*(*T*) is the PL intensity at a given temperature, *T*.

### 4.3. 2-Inch wafer samples for optimization of the growth conditions

We here provide the information that we can obtain the dense and homogeneous NWs at adequate growth window as shown in Fig. 1–3. Fig. 5 shows 2-inch wafer samples for optimization of the growth conditions. Substrates in Fig. 5(a), (b) and (j) were cleaved after the growth process for characterization by SEM and PL measurements. After nanowire growth, all the samples lost the mirror-like shiny surface observed for the pristine Si substrate, as shown in Fig. 5(k). Samples with a matte appearance, as shown in Fig. 5(a)–(c) and (h)–(j), had much less or even negligible nucleation of the nanowires than the samples with dense nanowires on the surface shown in Fig. 5(d)–(g). The samples in Fig. 5(d)–(g) have nucleation of the nanowires with densities higher than the order of 10^7^ cm^−2^. From examination of these growth conditions, the substrate temperature and the rate of flux and V/III ratio at the initial growth stage were determined as factors to obtain dense nanowires over the substrate. A sample with dense nanowires over the 2-inch substrate was then obtained at a Ga flux at 6.3 × 10^14^ cm^−2^ s^−1^, an atomic Ga : As_4_ flux ratio of 1, and a substrate temperature of 500 °C.

### 4.4. XRD characteristics

**Fig. 6:**
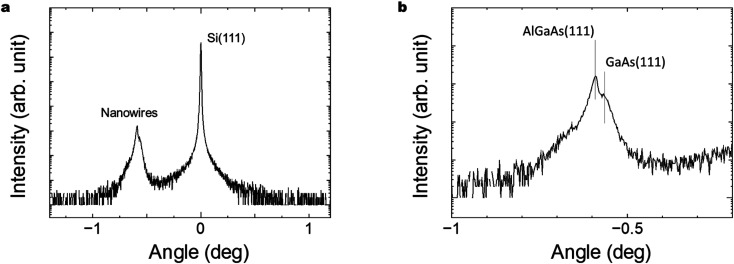
(a) *θ*–2*θ* XRD scan of the GaAs/Al_0.8_Ga_0.2_As core–shell nanowires investigated, and (b) enlarged curve for the peak originating from the nanowires. The centers of the curves were adjusted relative to the Si(111) reflection peak at 14.22°.

Fig. 6(a) and (b) show XRD characteristics of the GaAs/Al_0.8_Ga_0.2_As core–shell nanowires sample on Si(111) substrate. X-ray diffraction (XRD) measurements were conducted with a Malvern Panalytical X'Pert MRD system equipped with a graded parabolic X-ray mirror with a four-bounce Ge(220) monochromator and Cu Kα_1_ radiation. Symmetric *θ*–2*θ* scans were performed with a three-bounce Ge(220) analyzer and a Xe proportional detector around the Si(111) reflection. The clearly observed peaks related to the Si(111) substrate and nanowires close to GaAs(111) plane indicate the formation of crystalline GaAs nanowires on the Si substrate. Peaks originating from the GaAs-related nanowires were observed at −0.59° and −0.57° and are assigned to the zinc blende AlGaAs(111) and GaAs(111) planes, respectively.^47^ The NWs are hence predominantly zinc blende structures.^48^ The results indicate the presence of a larger volume AlGaAs shell compared to the inner GaAs core, as designed for the structure.

### 4.5. SEM-EDS observation

**Fig. 7:**
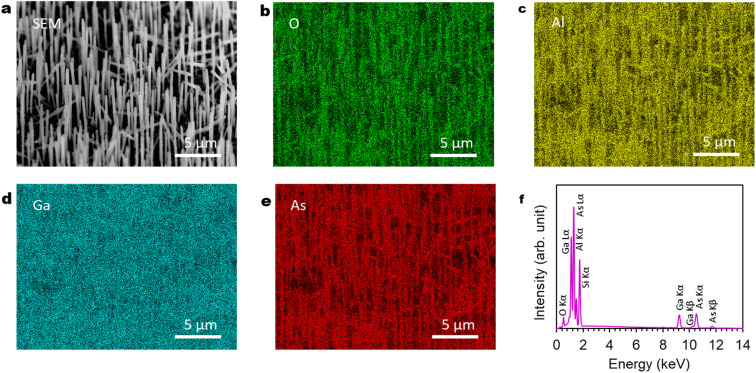
SEM and EDS results for GaAs/Al_0.8_Ga_0.2_As core–shell nanowires: (a) 45°-tilted SEM image. (b)–(e) EDS elemental mapping images of O, Al, Ga, As. (f) EDS spectra from the imaging area. The images were obtained with an acceleration voltage of 25 kV.

Fig. 7(a)–(f) show SEM-EDS observation of the GaAs/Al_0.8_Ga_0.2_As core–shell nanowire sample. The SEM image in Fig. 7(a) shows good agreement with the EDS images of O, Al, and As shown in Fig. 7(b)–(d). This confirms the presence of nanowires with large fractions of O, Al, and As elements as constituents, which is in agreement with the XRD results in Fig. 6. The spectrum in Fig. 7(f) shows peaks related to conceivable elements of O, Al, Si, Ga, and As from the GaAs/Al_0.8_Ga_0.2_As core–shell nanowires on the Si(111) substrate, which suggests the successful formation of the designed structure.

### 4.6. Axial cross-sectional STEM and EDS images of another nanowire

**Fig. 8:**
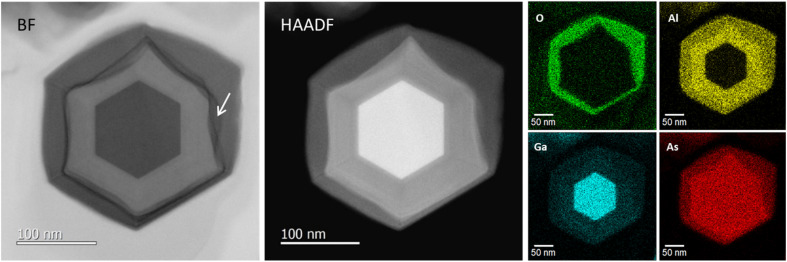
Axial cross-sectional BF-STEM and HAADF-STEM images of another typical nanowire with EDS elemental mapping for O, Al, Ga, and As for the identical GaAs/Al_0.8_Ga_0.2_As core–shell nanowire sample shown in Fig. 2.

Fig. 8 shows axial cross-sectional STEM and EDS images of another nanowire shown in Fig. 2. The variation of the individual nanowire structure was investigated here. (We refer to this nanowire as NW2 for the following discussion in Fig. 8.) The nanowire shows identical characteristics as those observed for the nanowire in Fig. 2. The nanowire has a regular hexagonal GaAs core with a 120 nm diameter enclosed by a 60 nm thick Al_0.8_Ga_0.2_As shell. The surface of the outermost AlGaAs shell was oxidized to form an AlGaO_*x*_ outer shell, as indicated by the EDS elemental maps. A dark area is observed at the interface between the initial AlGaAs shell and the oxidized AlGaO_*x*_, as indicated by the arrow in the BF image. Even though the extent of oxidation from the surface is different from the nanowire shown in Fig. 2(c)–(e) (the nanowire is referred to as NW1 for the discussion in Fig. 8), the inside of the AlGaAs shell and the inner GaAs core showed negligible oxide concentrations. These results indicate that the inner core and the inside of the AlGaAs are not affected by oxidation from the surface, and the crystalline quality is preserved.

### 4.7. Variation of individual nanowire structures

**Fig. 9:**
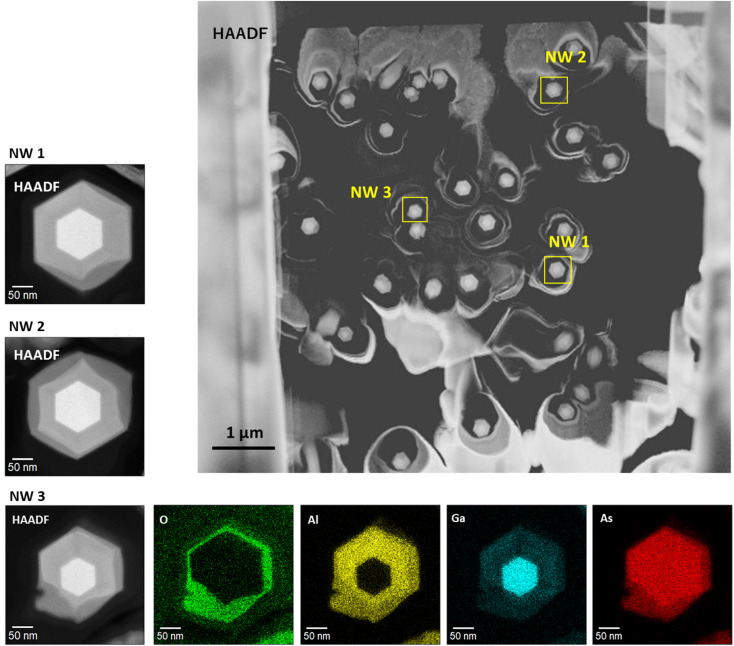
Variation of individual nanowire structures within the axial cross-sectional STEM specimen. NW1 is the wire shown in Fig. 2(c)–(e). NW2 is the wire shown in Fig. 8. NW3 was also selected as an example of a deteriorated structure within the sample.

Fig. 9 summarizes the variation of individual nanowire structures within the axial cross-sectional STEM specimen. In the figure, we show an HAADF-STEM image of the axial-cross-sectional specimen (upper right), HAADF images of NW1, NW2, and NW3, and EDS elemental maps for O, Al, Ga, and As for the NW3 GaAs/Al_0.8_Ga_0.2_As core–shell nanowires in the lower panels. From the specimen, we can observe numbers of nanowires, most of which have hexagonal cross-sectional structures. The enlarged HAADF images for NW1 and NW2 indicate the formation of the desired GaAs/AlGaAs core–shell structure with surface oxide, which was discussed in Fig. 2 and 8. The variation of the oxide extent from the surface of NW3 occasionally deteriorates the nanowire structure, and even cracks are formed from the surface. Nevertheless, the inner GaAs core and inside AlGaAs/GaAs interface were preserved without oxidation. This feature results in homogeneous PL over the substrate, which is measured with a laser having a spot diameter of *ca.* 100 μm.

### 4.8. Radial cross-sectional STEM-EDS characteristics

**Fig. 10:**
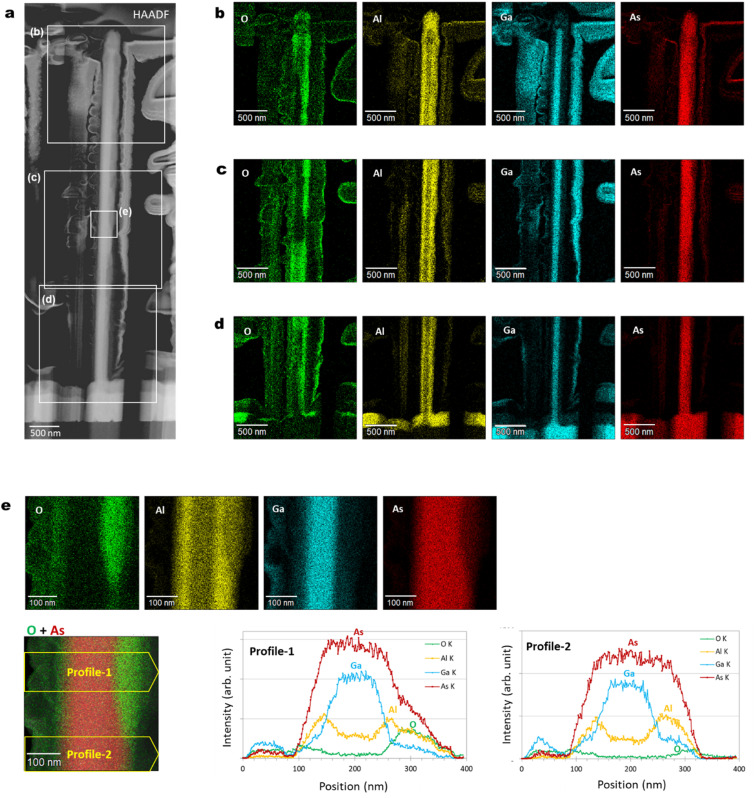
(a) BF-STEM image, and (b)–(d) EDS elemental maps of O, Al, Ga, and As for the areas indicated by the squares in (a) for a single GaAs/Al_0.8_Ga_0.2_As core–shell nanowire. (e) Enlarged EDS elemental maps for the areas indicated by the squares in (a), and EDS elemental distributions along the profiles through the nanowire shown in the superimposed elemental maps for O and As in the bottom left panel.

Fig. 10(a)–(d) show radial cross-sectional STEM-EDS characteristics of a GaAs/Al_0.8_Ga_0.2_As core–shell nanowire. The distribution of the oxide intensity observed in the elemental map of O in Fig. 10(b)–(d) is inhomogeneous, in contrast to the other constituent elements. This is probably an inherent characteristic of the native oxide, which occasionally occurs at the surfaces of nanowires, and this would also induce the variation of the axial cross-sectional structure shown in Fig. 9. Strong intensity with a sharp structured image was observed from the elemental mapping of Ga in Fig. 10(b)–(d). Fig. 10(e) shows EDS elemental map and profile for the area indicated in the middle part of the nanowires shown in Fig. 10(a). The Ga intensity originates from the GaAs core with a diameter of 120 nm, as designed. The distribution at the nanowire core shows a wider diameter than Ga; therefore, variation of its width is dependent on the progress of the oxides from the outer surface. The intensity profiles clearly indicate the preserved original structures of the inner GaAs core and GaAs/AlGaAs interface without any effect from the native oxides.^27,49^

### 4.9. Optical characteristics, reproducibility, and the effect of cleavage

**Fig. 11:**
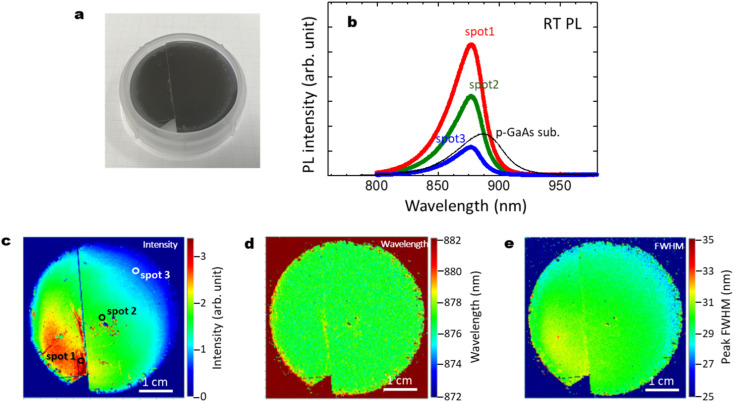
(a) Photograph of a 2-inch Si wafer after the growth of GaAs/Al_0.8_Ga_0.2_As core–shell nanowires; another sample different from that in Fig. 3(a). (b) RT PL intensity for the GaAs/Al_0.8_Ga_0.2_As core–shell nanowires measured at the spots on the substrate surface shown in (c), and for GaAs nanowires without the shell layers, and a commercial p-type GaAs substrate as a reference. (c)–(e) PL mapping results for the GaAs/Al_0.8_Ga_0.2_As core–shell nanowires with distributions of (c) intensity, (d) wavelength, and (e) FWHM. As shown in (a) and (c), cleavage does not affect the PL features.

Fig. 11(a)–(e) summarize the optical characteristics, reproducibility and the effect of cleavage for another 2-inch wafer sample with those shown in Fig. 3. The GaAs/Al_0.8_Ga_0.2_As core–shell nanowire sample showed strong PL intensity with a peak wavelength at 876 nm. The p-type GaAs substrate produced a peak at 884 nm due to the band gap shrinkage induced by its carrier concentration at 2 × 10^19^ cm^−3^.^50^ The intensity of the p-type GaAs substrate shows comparable or weaker intensity than the GaAs/Al_0.8_Ga_0.2_As core–shell nanowire sample. The slightly shorter peak wavelength for the passivated nanowires compared to that for the p-GaAs substrate is considered to be due to compressive strain induced by the surface oxides.^27,49^ As shown in Fig. 3(c), the PL intensity varies across the surface, where most of the area has stronger intensity than the p-GaAs substrate. As seen in the PL mapping results shown in Fig. 11(c)–(e), cleavage does not affect the PL features. The distribution of the PL peak wavelength was homogeneous over the sample, with a peak wavelength of 878 nm and a standard deviation of 3 nm. The peak FWHM was 30 nm with a standard deviation of 2 nm. This highly homogeneous feature is desirable for practical applications, and it is reproducible, as shown in Fig. 3.

### 4.10. Temperature dependence of PL emission properties

**Fig. 12:**
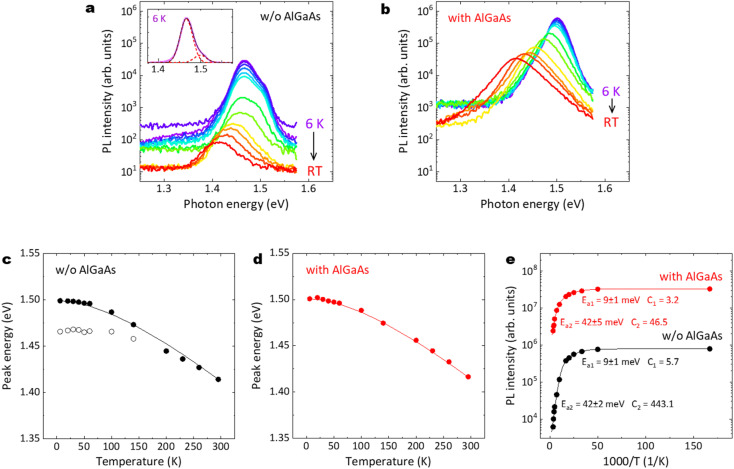
PL spectra at temperatures from 6 K to RT for the nanowires (a) without and (b) with the AlGaAs shell. The inset in (a) shows the double-peak fitting of the PL spectrum at 6 K. PL peak energies as a function of temperature for the nanowires (c) without and (d) with the AlGaAs shell. The solid lines show the fits to these data using Varshni's equation with the appropriate parameter for GaAs. (e) Integrated near-GaAs-band-edge PL intensity as a function of inverse temperature with Arrhenius fits (solid lines). As shown in (a), two major peaks can be observed for the nanowires without the AlGaAs shell.

Fig. 12(a)–(e) summarize the temperature dependence of PL emission properties for the GaAs/Al_0.8_Ga_0.2_As core–shell nanowires. As shown in Fig. 12(a), two major peaks can be observed for the nanowires without the AlGaAs shell. The small peak at 1.50 eV corresponds to exciton emission in GaAs. The peak at 1.465 eV may be due to an impurity-related emission. This lower-energy emission is strongly quenched with increasing temperature and almost disappears above 140 K, whereas the higher-energy emission remains up to RT and its peak energy follows Varshni's equation, as shown with the solid line in Fig. 12(c).^51^ In contrast, for the nanowires with the AlGaAs shell, a single peak that corresponds to band edge transitions can be observed, regardless of temperature, as shown in Fig. 12(b). The peak energy follows the Varshni equation well, as shown with the solid line in Fig. 12(d). The temperature dependence of the near-GaAs-band-edge PL intensity is plotted in Fig. 12(e). These plots can be well fitted with the well-known Arrhenius expression, *I*(*T*) = *I*_0_/(1 + *C*_1_ exp(−*E*_a1_/*k*_B_*T*) + *C*_2_ exp(−*E*_a2_/*k*_B_*T*)), where *I*(*T*) is the integrated PL intensity at a given temperature *T*. *I*_0_ is the integrated PL intensity when the temperature approaches 0 K. *E*_a1_ and *E*_a2_ represent the activation energy for the thermal quenching process at low and high temperatures, respectively. *C*_1_ and *C*_2_ are the coefficients of these quenching efficiencies and *k*_B_ is the Boltzmann constant. For both nanowire samples, the same *E*_a1_ and *E*_a2_ were obtained; activation energies of *E*_a1_ = 9 meV and *E*_a2_ = 42 meV were obtained at low and high temperatures, respectively. The first quenching channel can be derived from the dissociation of free excitons or a thermally activated delocalization of excitons from the minima of potential fluctuations.^52^ The second quenching channel can be mainly attributed to non-radiative surface recombination, which is dominant at high temperatures. The *C*_2_ value for the nanowires with the AlGaAs shell is one order of magnitude smaller than that without the AlGaAs shell, which clearly demonstrates that the AlGaAs shell can provide efficient passivation for the nanowires to realize a highly efficient IQE at RT.

### 4.11. Time-resolved PL and internal quantum efficiency

**Fig. 13:**
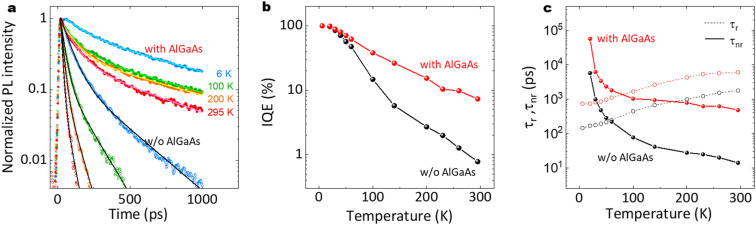
(a) Normalized PL decay curves with biexponential decay fitting lines (solid lines) at different temperatures, (b) IQE, and (c) radiative and non-radiative recombination lifetimes as a function of temperature for the nanowires with and without the AlGaAs shell, different from that in Fig. 4(a)–(c).

Fig. 13(a)–(c) show time-resolved PL results and the estimated internal quantum efficiency (IQE). As shown in Fig. 13(a), the AlGaAs shell passivation efficiently extended the carrier lifetime. The average lifetime for the passivated nanowires at RT was 424 ps, although that for the unpassivated nanowires was 13 ps. Note that the passivated sample showed a slower decay of *ca.* 1 ns after 500 ps, which is comparable to the long carrier lifetime reported for the GaAs/AlGaAs system.^53^ As shown in Fig. 13(b), the passivated sample preserved the IQE at larger than 10% up to RT, compared to the IQE of 1.6% at RT for the unpassivated sample. The radiative and non-radiative recombination times are summarized in Fig. 13(c). The passivated nanowires showed a non-radiative lifetime of 473 ps, which was 36 times longer than that for the unpassivated nanowires (13 ps). These results clearly demonstrate that the AlGaAs and oxide shell provide efficient passivation for the nanowires to realize a highly efficient IQE at RT.

## Data availability

The data that support the findings of this study are available from the corresponding author upon reasonable request.

## Author contributions

Sample growth, and structural and optical investigations were carried out by K. M., R. M., H. H., K. N., K. S., R. T., T. T., and M. Y. K. N. and T. Y. conducted SEM and EDS measurements and discussed the structure of the nanowires. S. S., S. H., and A. M. performed time-resolved PL measurements and investigated the carrier dynamics. All the authors discussed the results. F. I. designed the project and wrote the final version of the manuscript with feedback from all the co-authors.

## Conflicts of interest

The authors declare no competing interests or personal relationships that could have appeared to influence the work reported in this paper.

## Supplementary Material
